# Renal prognostic value of serum monoclonal immunoglobulin in cryoglobulinemic glomerulonephritis

**DOI:** 10.3389/fimmu.2025.1578295

**Published:** 2025-07-29

**Authors:** Lei Ma, Yuanyuan Xia, Yun Fan, Dan Zhou, Xinchen Yao, Yongzhong Zhong, Fan Yang, Feng Xu, Shaoshan Liang, Yujie Wang, Xiaodong Zhu, Dacheng Chen, Rong Tan, Zhengyun Zhu, Dandan Liang, Caihong Zeng

**Affiliations:** ^1^ National Clinical Research Center for Kidney Diseases, Jinling Hospital, Affiliated Hospital of Medical School, Nanjing University, Nanjing, China; ^2^ The Affiliated Wuxi People’s Hospital of Nanjing Medical University, Wuxi People’s Hospital, Department of Nephrology, Wuxi Medical Center, Nanjing Medical University, Wuxi, China; ^3^ National Clinical Research Center for Kidney Diseases, Jinling Hospital, Nanjing Medical University, Nanjing, China

**Keywords:** clinicopathological features, monoclonal immunoglobulin, cryoglobulinemic glomerulonephritis, prognostic factors, macrophage - cell

## Abstract

**Aims:**

To explore the clinicopathological features and renal outcome in patients with cryoglobulinemic glomerulonephritis (Cryo-GN) without confirmed systemic autoimmune diseases.

**Methods:**

Sixty-nine patients with Cryo-GN from a single center were recruited in this retrospective study. Their clinical, pathologic, and follow-up data were collected and analyzed. According to whether the serum monoclonal immunoglobulin (MIg) and HBV-DNA/HBV markers or HCV-RNA/anti-HCV antibodies were positive or not, they were classified into four groups: positive serum MIg only (MIg group), positive HBV-DNA/HBV markers or HCV-RNA/anti-HCV antibodies (HBV/HCV) only (HBV/HCV group), positive serum MIg and HBV/HCV (MIg+HBV/HCV group), and all MIg/HBV/HCV negative group.

**Results:**

The male-to-female ratio was 1.38:1 with a mean age of 50.4 ± 14.7 years in the patient cohort. Hypertension was presented in 59.4% of cases, anemia in 73.9%, renal insufficiency in 60.9%, nephrotic proteinuria in 44.9% and microscopic hematuria in 94.2%. The MIg group had significantly lower eGFR levels, higher cryoglobulin levels, and higher rates of abnormal serum-free light chain ratios than the MIg/HBV/HCV negative group. The most common histological pattern of Cryo-GN was membranoproliferative glomerulonephritis (MPGN), and the MIg group had significantly higher scores of the severity of intracapillary cryo-Plugs than the MIg+HBV/HCV group and the MIg/HBV/HCV negative group. Immunohistochemical staining of 29 patients revealed a significant infiltration of CD68+ cells within the glomeruli. Further multiplex immunohistochemical staining of 4 of these patients showed that the infiltrating cells within the glomeruli in Cryo-GN were predominantly CD68+CD163+ cells. Sixty-seven patients had a median follow-up of 31.7 months, and 23.9% of them progressed to end-stage renal disease (ESRD). The renal survival was inferior for MIg group than HBV/HCV group. Multivariate analysis showed that serum MIg and eGFR were independent prognostic factors.

**Conclusion:**

Regardless of the presence of HBV/HCV infection, non-systemic autoimmune diseases related Cryo-GN patients with serum MIg had worse renal function and renal survival. Patients with a large number of pseudothrombi in the glomerular capillary lumens tend to have worse renal outcomes. Serum MIg and eGFR were independent risk factors for renal survival in Cryo-GN patients without autoimmune diseases.

## Graphical Abstract

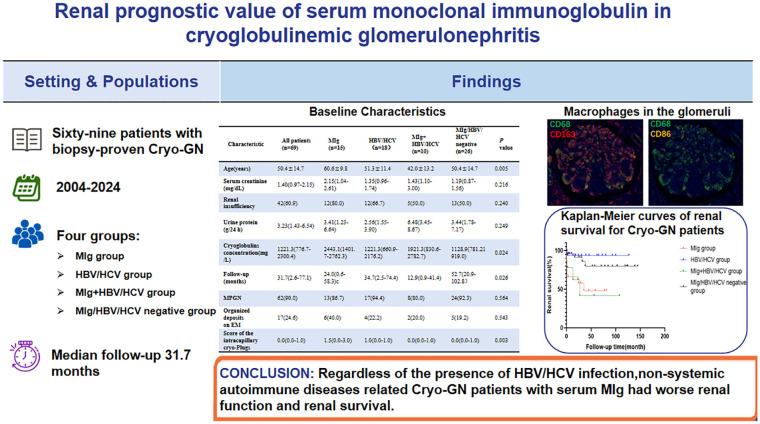

## Introduction

Cryoglobulins are serum immunoglobulins that precipitate at temperatures below 37°C and redissolve on warming. Cryoglobulinemia is divided into three types based on immunoglobulin composition: type I (composed of a single monoclonal immunoglobulin, typically associated with lymphoproliferative disorders such as multiple myeloma or Waldenström macroglobulinemia), type II (mixed cryoglobulins consisting of monoclonal IgM with rheumatoid factor activity and polyclonal IgG, most commonly linked to hepatitis C virus infection), and type III (polyclonal IgM and IgG, often related to chronic infections or autoimmune diseases) (1, 2). Precipitating in the microcirculation and immune-complex-mediated inflammation of blood vessels are the two major pathogenic mechanisms of cryoglobulins. The skin, kidneys, nervous system, and joints can be involved. Cryoglobulinaemia is associated with many diseases, which can be generally grouped into infections, autoimmune disorders, and malignancies (2, 3).

Kidney is one of the most involved organs in cryoglobulinemia, named as cryoglobulinemic glomerulonephritis (Cryo-GN) (2.3). Cryo-GN is a recognized independent risk factor of the poor prognosis of cryoglobulinemia (4–6). Several studies have indicated that 9-14% of patients with Cryo-GN may progress to end-stage renal disease (ESRD) (7–10). Previous studies had shown that hepatitis B virus (HBV) infection is more common than hepatitis C virus (HCV) infection among Chinese patients with cryoglobulinemia, and there is relatively less research on HBV-related cryoglobulinemic glomerulonephritis (Cryo-GN) (11, 12). According to the new consensus on monoclonal gammopathy of renal significance, some type I and type II cryoglobulinemia-related glomerulonephritis are classified as monoclonal gammopathy of renal significance (MGRS)-related diseases (13). However, the differences in clinical and renal histopathological features of cryoglobulinemia caused by different etiologies have not been fully elucidated. This study retrospectively analyzed 69 Chinese patients who had biopsy-proven Cryo-GN and explored the clinical and pathological differences of Cryo-GN caused by different etiologies, to deepen the understanding of Cryo-GN, improve the clinical diagnosis, treatment level, and the prognosis of patients.

## Materials and methods

### Patient selection

**Figure 1:**
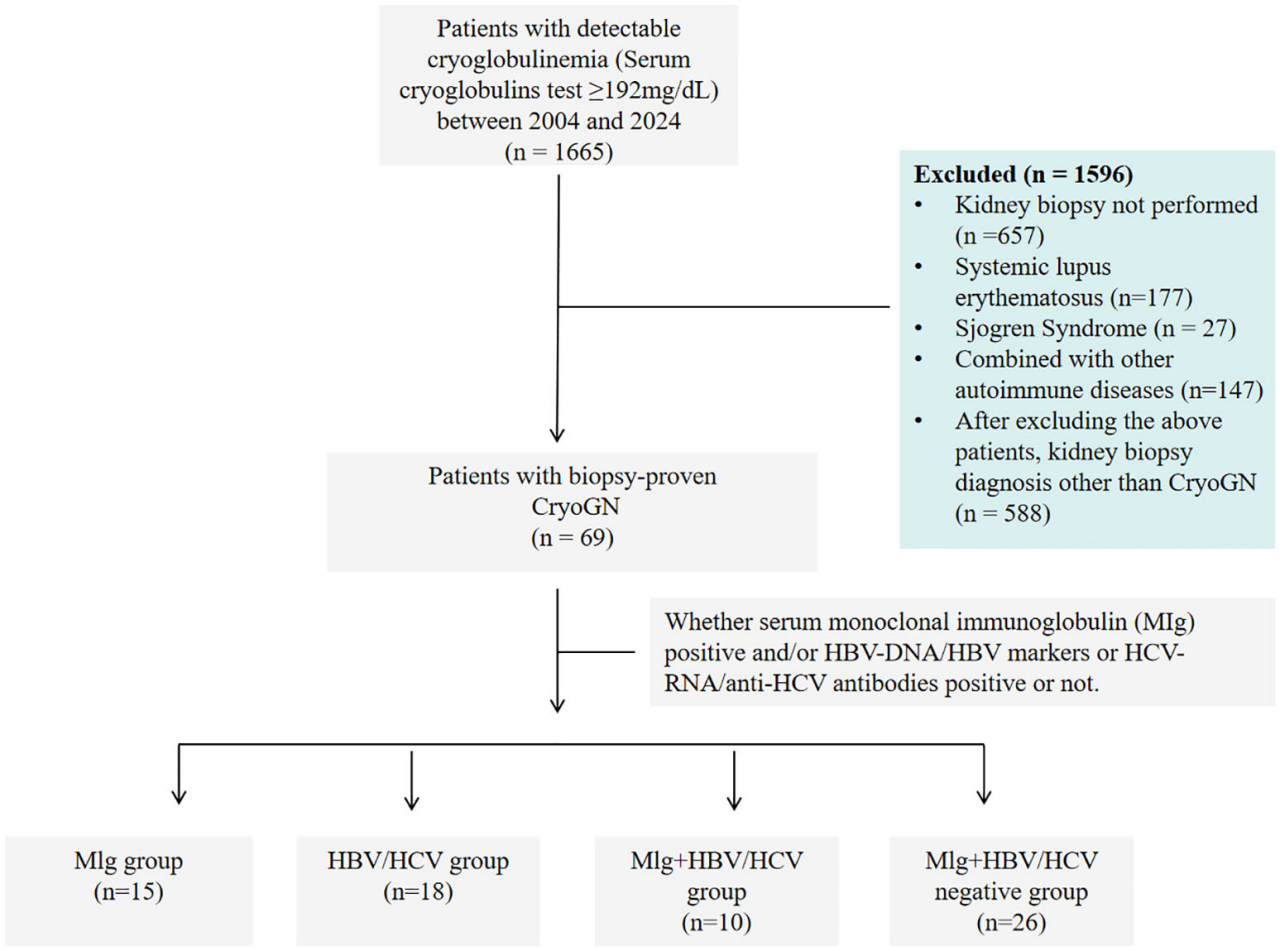
Study flow chart of patients with cryoglobulinemia who underwent a kidney biopsy from 2004 to 2024.

Sixty-nine patients with Cryo-GN were identified by retrospective review of all native renal biopsies received at the National Clinical Research Center for Kidney Diseases, Jinling Hospital, from January 2004 to December 2024 (**Figure 1**). Inclusion criteria were (1): Serum cryoglobulins test ≥192mg/dL (2); Accompanied by clinical manifestations of renal damage such as proteinuria, hematuria, and renal dysfunction (3); Kidney biopsy findings showing MPGN or endocapillary proliferative glomerulonephritis patterns excluding other defined causes such as acute poststreptococcal glomerulonephritis, lupus nephritis, IgA nephropathy, C3 glomerulopathy, monoclonal immunoglobulin deposition, and genetic glomerulonephritis. We excluded patients diagnosed with autoimmune conditions, including systemic lupus erythematosus, rheumatoid arthritis, or Sjögren’s syndrome, from subsequent analyses owing to their distinctive disease mechanisms, therapeutic regimens, and clinical outcomes.

According to whether serum monoclonal immunoglobulin (MIg) was positive and/or HBV-DNA/HBV markers or HCV-RNA/anti-HCV antibodies positive or not, all participants were classified into four groups: Only positive serum MIg (MIg group), only positive for HBV-DNA/HBV markers or HCV-RNA/anti-HCV antibodies (HBV/HCV group), both positive for MIg and HBV/HCV (MIg+HBV/HCV positive group), both negative for MIg and HBV/HCV (MIg/HBV/HCV negative group).

Patient’s medical records were reviewed for demographic information, clinical and laboratory data at the time of biopsy, treatment and follow-up. The cause of cryoglobulinemia was established according to the research of Brouet et al (1).

The clinical manifestations were defined as follows: hypertension: systolic blood pressure >140 mmHg or diastolic blood pressure>90mmHg or ongoing use of antihypertensive medications; anemia: hemoglobin (Hb) <120 g/L in males, Hb <110 g/L in females (14); nephrotic-range proteinuria (NRP): proteinuria≥3.5g/24h; microscopic hematuria: urinary sediment erythrocyte count > 12/μL(SysmexUF-1000 urine analyzer); renal insufficiency: estimated glomerular filtration rate (eGFR) (EPI)<60 mL/min/1.73 m² for ≥3 months; abnormal serum free light chain (FLC) ratio: κ/λ<0.26 or>1.65(Freelite, BindingSite, UK); RF positive (RF > 20 (IU/ml). ESRD: eGFR (EPI)<15ml/(min.1.73m2) or being dependent on renal replacement therapy (15). The serum anti-HCV antibody, HBV markers, HCV-RNA, and HBV-DNA were also obtained. Definition of renal response to treatment (10): Complete remission (CR) (1): Urinary protein <0.4 g/24 h (2), Disappearance of hematuria (3). When the baseline eGFR is lower than 70 ml/(min.1.73m2), the eGFR after treatment increases by 20% compared with the baseline level. Partial remission (PR): At least one of the criteria for complete remission is met. No response (NR): None of the criteria for complete remission is met. Follow-up endpoints: progress to ESRD (estimated glomerular filtration rate(eGFR)(EPI)<15ml/(min.1.73m^2^) or being dependent on renal replacement therapy).

### Detection of cryoglobulins

(1) Syringes and test tubes were prewarmed to 37°C. 15ml of venous blood were collected into warmed tubes, 37°C water bath until clotted (2). After the separation of serum, serum was centrifuged at 37°C, 2000rpm (3). 5ml of supernatant was accurately collected and stored at 4°C for precipitation for 5–7 days (4). After precipitation and centrifugation, cryoprecipitate was isolated and washed with cold PBS (< 4°C) for 3 times (5). After dissolution in 3 mL sodium acetate buffer, the absorbance was measured at OD280 and OD260 with a microplate reader. Cryoglobulin concentration =1550×OD280- 770×OD260. The cryoglobulin detection method in this study has a sensitivity of 0.1–0.5 mg/mL and a positive threshold set at 0.192 mg/mL (192 mg/L), it can effectively identify clinically significant cryoglobulinemia.

### Pathology studies

All renal biopsies were processed using the standard techniques of light microscopy (LM), immunofluorescence (IF), and electron microscope (EM). Acute tubular injury was defined by the presence of tubular simplification, loss or attenuation of the brush border. Interstitial fibrosis/tubular atrophy (IF/TA) and acute tubular injury (ATI) were scored semiquantitatively based on the percentage of the tubulointerstitial compartment affected and recorded as 0% (0), 1%-25% (1), 26%-50% (2), or >50% (3). The severity of intracapillary thrombi in glomeruli was scored according to the proportion of glomeruli affected: 0 (0), <25% (1); 25-75% (2); >75% (3). IF was performed on cryosections (4μm) using polyclonal FITC-conjugated antibodies against IgG, IgM, IgA, C3, C1q, κ, and λ light chains (Dako Corp., Glostrup, Denmark). Cases with IgG deposits underwent IgG subtype staining. Ultrastructural evaluation was performed using a FEI Tecnai G2 Spirit transmission electron microscope.

### Immunohistochemical staining

Formalin-fixed, paraffin-embedded kidney tissue sections were processed according to routine clinical practice. Kidney tissue sections were stained for CD3 (Clone 565, Newcastle, 1:100) as a T-cell marker, CD4 (Clone L26, Dako) as a CD4+ T-cell marker, and CD8 (Clone L26, Dako) as a CD8+ T-cell marker. The staining was performed on consecutive sections of patient renal tissue using a standard automated immunostaining device (Leica BOND, United States). Positive cell counts were determined for tangential and non-sclerotic glomeruli.

### Multiplex immunohistochemical staining

Cryopreserved 3.5-μm paraffin tissue sections from renal biopsy specimens were sequentially placed into xylene I for 10 min, xylene II for 10 min, xylene III for 10 min, anhydrous ethanol I for 5 min, anhydrous ethanol II for 5 min, 90% ethanol for 5 min, 70% ethanol for 5 min, and rinsed in distilled water for 3 min. Antigen retrieval was then performed using citrate buffer (pH 6.0) (Akoya Biosciences, AR600250ML). Endogenous peroxidase activity was blocked with a peroxidase-blocking solution (Beyotime Biotechnology, P0100B), followed by serum blocking with goat serum. The sections were incubated with a primary antibody or an isotype-matched control antibody at room temperature for 1 hour. After incubation with a universal secondary antibody for mouse and rabbit (Akoya Biosciences, ARH1001EA) at room temperature for 30 minutes, corresponding dyes were applied according to the primary antibody staining sequence (CD68 (Abcam, ab955, pan-macrophage marker), CD163 (Abcam, ab182422, M2 macrophage marker), CD86 (CST, 91882S, M1 macrophage/antigen-presenting cell marker), CD206 (CST, 24595S, M2 macrophage mannose receptor), CD56 (CST, 99746S), CD3 (Abcam, ab11089), CD8 (Abcam, ab199016)), and incubated for 10 min. Slides were then washed three times in TBST for 5 min each. The above steps were repeated according to the primary antibody staining sequence until all markers were completed. Subsequently, the slides were counterstained with DAPI, mounted, and imaged using a slide scanner. Using Qupath-0.5.0 software for image analysis, select a kidney tissue and count all types of marker cells within the glomeruli, taking the average and maximum values.

### Statistical analyses

Continuous variables are presented as the means ± SD and non-normally distributed data are expressed as the median(interquartile rang(IQR). Survival analysis was performed by univariate survival analysis, multivariable Cox regression models, and Kaplan–Meier curves. Most clinical variables are assessed in continuous ways while pathological parameters in categorical ways. Kruskal-Wallis test and nonparametric Mann–Whitney test were used for the comparison of different groups, followed by Dunn-Bonferroni *post hoc* test for pairwise comparisons. Statistical significance was assumed at *P*<0.05. All statistical analysis was a two-sided test. Statistical analysis was performed with SPSS (version 27.0, SPSS, Chicago, IL, USA).

## Results

### General demographics and clinical characteristics

The clinical characteristics of the patients at kidney biopsy are described in **Table 1**. There were 40 males and 29 females, mean age 50.4 ± 14.7 years. Twenty-one patients (30.4%) were positive for serum HBV markers or HBV-DNA and 7 patients (10.1%) were positive for anti-HCV antibodies or HCV-RNA. The median 24-h urine protein was 3.23g/d (1.43-6.54g/d) and 44.9% of patients had nephrotic proteinuria. Renal dysfunction was observed in 60.9% of patients, with a median serum creatine of 1.40mg/dL (0.97-2.15mg/dL). The median concentration of cryoglobulin in the 69 patients was 1221.3mg/L (776.7-2300.4mg/L). Sixty-four patients underwent routine RF testing, with a median RF level of 128.5 IU/ml (19.9-555.0 IU/ml).

**Table 1:** Demographic and clinical data in Cryo-GN patients without autoimmune diseases at renal biopsy.

Characteristic	All patients (n=69)	MIg (n=15)	HBV/HCV (n=18)	MIg+ HBV/HCV (n=10)	MIg/HBV/HCV negative (n=26)	*P* value
Sex (M/F)	40/29	10/5	10/8	6/4	14/12	0.896
Age (years)*	50.4 ± 14.7	60.6 ± 9.8^b^ ^c^	51.3 ± 11.4	42.0 ± 13.2	50.4 ± 14.7	0.005
Renal lesions duration (months)^#^	7.0 (1.5-24.0)	7.0 (1.0-13.0)	12.0 (2.0-27.8)	18.0 (5.3-84.0)	3.5 (1.0-30.0)	0.262
Hypertension	41 (59.4)	13 (86.7)	9 (50.0)	6 (60.0)	13 (50.0)	0.102
Anemia	51 (73.9)	12 (80.0)	15 (83.3)	8 (80.00	16 (61.5)	0.384
HBV infection	21 (30.4)	0 (0.0)^abde^	13 (72.2)	8 (80.0)	0 (0.0)	<0.001
HCV infection	7 (10.1)	0 (0.0)	5 (27.7)	2 (20.0)	0 (0.0)	–
Hematological malignancy §	7 (10.1)	2 (13.3)	3 (16.7)	0 (0.0)	2 (7.7)	0.590
Purpura	12 (17.4)	2 (13.3)^e^	3 (16.7)	5 (50.0)	2 (7.7)	0.036
Arthralgia	7 (10.1)	1 (6.7)	0 (0.0)	2 (20.0)	4 (15.4)	0.215
Renal insufficiency	42 (60.9)	12 (80.0)	12 (66.7)	5 (50.0)	13 (50.0)	0.240
Nephrotic proteinuria	31 (44.9)	7 (46.7)	5 (27.8)	7 (70.0)	12 (46.2)	0.203
Microscopic hematuria	65 (94.2)	15 (100.0)	17 (94.4)	10 (100.0)	23 (88.5)	0.510
Serum creatinine (mg/dL)^#^	1.40 (0.97-2.15)	2.15 (1.04-2.61)	1.35 (0.96-1.74)	1.43 (1.10-3.00)	1.19 (0.87-1.56)	0.216
eGFR (ml/min per 1.73m^2^)^#^	49.0 (29.0-76.6)	28.4 (24.1-51.5)^c^	54.0 (35.2-95.5)	67.0 (23.1-78.6)	61.3 (37.9-84.7)	0.044
Urine protein (g/24 h) ^#^	3.23 (1.43-6.54)	3.41 (1.23-6.64)	2.56 (1.55-3.90)	6.48 (3.45-8.67)	3.44 (1.78-7.17)	0.249
Cryoglobulin concentration (mg/L)^#^	1221.3 (776.7-2300.4)	2443.1 (1401.7-2762.3)^c^	1221.3 (660.9-2176.2)	1921.3 (830.6-2782.7)	1128.9 (781.2-1919.0)	0.024
Abnormal serum FLC ratio	34 (50.7)	11 (88.4)^c^ ^f^	8 (44.4)	8 (80.0)	7 (26.9)	0.001
Decrease of C3 level	47 (68.1)	11 (73.3)	6 (33.3)	6 (60.0)	14 (53.8)	0.085
Decrease of C4 level	31 (44.9)	7 (46.7)^d^	12 (66.7)	6 (60.0)	6 (23.1)	0.023
Decrease of C3 and C4 levels	27 (39.1)	6 (40.0)^d^	12 (66.7)	4 (40.0)	5 (19.2)	0.017
RF positive	46 (71.9)	7 (46.7)	14 (82.4)	8 (88.9)	17 (73.9)	0.102
Follow-up (months)^#^	31.7 (2.6-77.1)	24.0 (0.6-58.3)^c^	34.7 (2.5-74.4)	12.9 (0.9-41.4)	52.7 (20.9-102.8)	0.026

*Data are shown as mean ± SD. ♯: Data are given as median (interquartile range). The remaining data are given as number (percentage). §, Bone marrow biopsy shows hematologic malignancy in 10cases, including B-cell lymphoma (n=5), lymphoplasmacytic lymphoma (n=1) and 4 cases not being excluded from B lymphoproliferative disorders.

a MIg vs HBV/HCV, *P*<0.05.

b MIg vs MIg+ HBV/HCV, *P*<0.05.

c MIg vs MIg/HBV/HCV negative, *P*<0.05.

d HBV/HCV vs MIg+HBV/HCV, *P*<0.05

e HBV/HCV vs MIg+HBV/HCV negative, *P*<0.05.

f MIg+HBV/HCV vs MIg/HBV/HCV negative, *P*<0.05.

Serum immunofixation electrophoresis (SIFE) detection was performed in all patients, and 25 cases (35.0%) were positive, of which 15 were IgM κ (60.0%), 4 were IgG κ (16.0%), 3 were IgG λ (12.0), 2 were κ alone and 1 were λ alone. A total of 66 patients underwent the detection of serum free light chain κ and λ, of which 34 (50.7%) had an abnormal ratio. Forty-three cases underwent bone marrow biopsy after admission, and 5 cases showed hematologic malignancy including 2 diffuse large B-cell lymphoma, 2 small B-cell lymphoma, 1 lymphoplasmacytic lymphoma. Besides, 4 patients showed B-lymphoproliferative disorder and 1 Plasmacytoplastic lesions by bone marrow biopsy. Two cases were diagnosed with B cell lymphoma prior to admission (MIg/HBV/HCV negative group).

### Clinical data in different groups

In this study, all patients were classified into four groups, 15 in MIg group, 18 in HBV/HCV group, 10 in MIg+HBV/HCV positive group, and 26 in MIg/HBV/HCV negative group. The clinical data of different groups are summarized in **Table 1**. The level of cryoglobulin and the rate of abnormal serum FLC ratio in MIg group were significantly higher than those in MIg/HBV/HCV negative group (P<0.05). Besides, the eGFR level and the renal survival time in MIg group were lower than those in MIg/HBV/HCV negative group (P<0.05).

### Pathologic characteristics

#### Light microscopy

**Figure 2:**
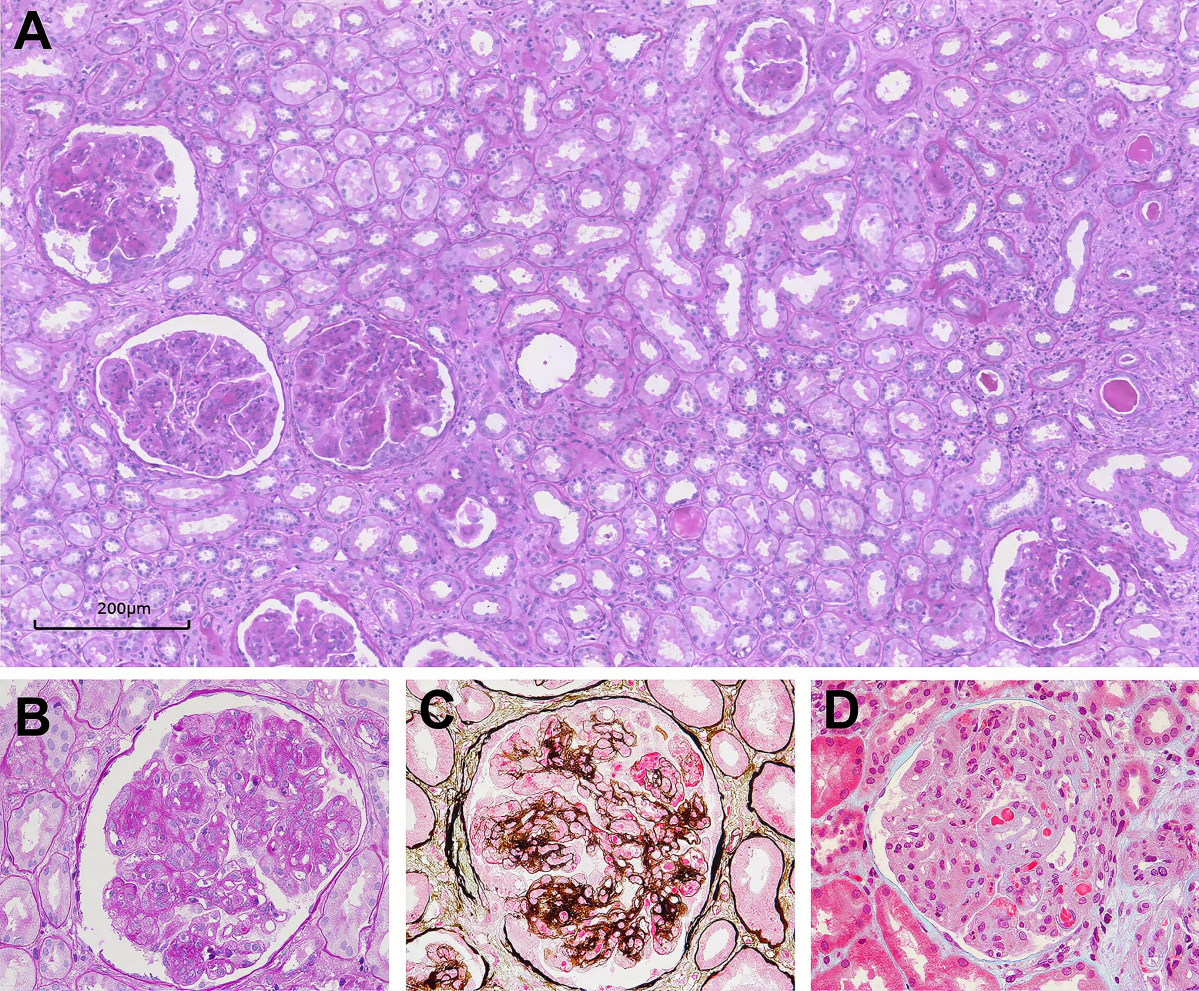
Glomerular lesions in Cryo-GN on light microscopy. **(A–D)** Light microscopy examination showing typical membranoproliferative glomerulonephritis (PAS, 100×, PAS, 400×, PASM-Masson, 400×, and Masson, 400× respectively).

All kidney biopsy samples were available for review (**Table 2**). The median number of glomeruli obtained by light microscopy was 27 (20–36). Twenty-eight patients (40.6%) had glomerular crescent formation. Membranoproliferative GN (MPGN) was the predominant histologic pattern, seen in 62 (90.0%) of cases (shown in **Figure 2**). Strongly PAS positive and fuchsinophilic/eosinophilic intracapillary cryo-plugs were observed in 42% of patients. The serum MIg-positive group had a significantly higher score of the intracapillary cryo-Plugs than the MIg+ HBV/HCV group and the MIg/HBV/HCV negative group.

**Table 2:** Histopathologic characteristics in Cryo-GN patients without autoimmune diseases.

Histopathologic characteristics	All patients (n=69)	MIg (n=15)	HBV/HCV (n=18)	MIg+HBV/HCV (n=10)	MIg/HBV/HCV negative (n=26)	*P* value
Histologic pattern						
MPGN	62 (90.0)	13 (86.7)	17 (94.4)	8 (80.0)	24 (92.3)	0.564
EPGN	6 (8.7)	2 (13.3)	1 (5.6)	2 (20.0)	1 (3.8)	
MsPGN	1 (1.4)	0 (0)	0 (0)	0 (0)	1 (3.8)	
Light microscopy						
Globally sclerotic glomeruli	50 (72.4)	10 (66.7)	13 (72.2)	7 (70.0)	20 (76.9)	0.942
Score of acute tubular injury	1.0 (1.0-2.0)	1.0 (1.0-2.0)	1.0 (0.75-1.25)	1.0 (1.0-2.0)	1.0 (0.75-2.0)	0.531
Score of TA/IF	1.0 (1.0-1.0)	1.0 (0.75-1.25)	1.0 (0.0-1.0)	1.0 (0.75-2.0)	1.0 (1.0-1.0)	0.502
Score of the intracapillary Cryo-Plugs	0.0 (0.0-1.0)	1.5 (0.0-3.0)^bc^	1.0 (0.0-1.0)	0.0 (0.0-1.0)	0.0 (0.0-1.0)	0.003
Immunofluorescence						
Deposition of IgG IgA IgM	28 (40.6)	5 (33.3)	6 (33.3)	5 (50.0)	12 (46.2)	0.733
Deposition of IgG IgA	2 (2.9)	0 (0.0)	0 (0.0)	0 (0.0)	2 (7.7)	–
Deposition of IgG IgM	25 (36.2)	6 (40.0)	7 (38.9)	4 (40.0)	8 (30.8)	0.909
Deposition of IgM	9 (13.0)	2 (13.3)	4 (22.2)	1 (10.0)	2 (7.7)	0.570
Deposition of IgG	5 (7.2)	2 (13.3)	1 (5.6)	0 (0.0)	2 (7.7)	0.793
Organized deposits on EM	17 (24.6)	6 (40.0)	4 (22.2)	2 (20.0)	5 (19.2)	0.543

MPGN, Membranoproliferative Glomerulonephritis; EPGN, Endocapillary Proliferative Glomerulonephritis; IF, Immunofluorescence; EM, Electron Microscopy.

TA/IF, tubular atrophy and interstitial fibrosis.

b MIg vs MIg+ HBV/HCV, *P*<0.05.

c MIg vs MIg/HBV/HCV negative, *P*<0.05.

#### Immunofluorescence

Fifty-five out of 69 cases had multiple Igs deposition in kidney biopsy samples, 5 cases had IgG deposition only, and 9 cases had IgM deposition only (shown in **Supplementary Figure 1**, **Table 2**). There is positive staining for IgG in 60 patients. Positive staining for C3 was observed in 90.0% of the cases, whereas positive staining for C1q was reported in 53.6% of the cases. In 25 patients with serum MIg (the Mlg group and Mlg+HBV/HCV group), only 4 patients exhibited monoclonal immunoglobulin deposition in renal tissue. In 44 patients with negative serum MIg (the HBV/HCV and Mlg+HBV/HCV negative groups), 9 patients showed positive staining for immunoglobulin of single heavy chain class in renal tissue, including 2 patients with monoclonal immunoglobulin deposition. Seven patients showed single IgG subtype in glomeruli, 4 had IgG3 subtype and 3 had IgG1 subtype. Two patients with IgG3 subclass had single κ light chain positivity, while the remaining patients exhibited polyclonal immunoglobulin deposition. **Supplementary Table 1** shows the results of immunofluorescence in patients with serum MIg and/or single Ig or IgG subtype deposits in renal tissue.

#### Electron microscopy

**Figure 3:**
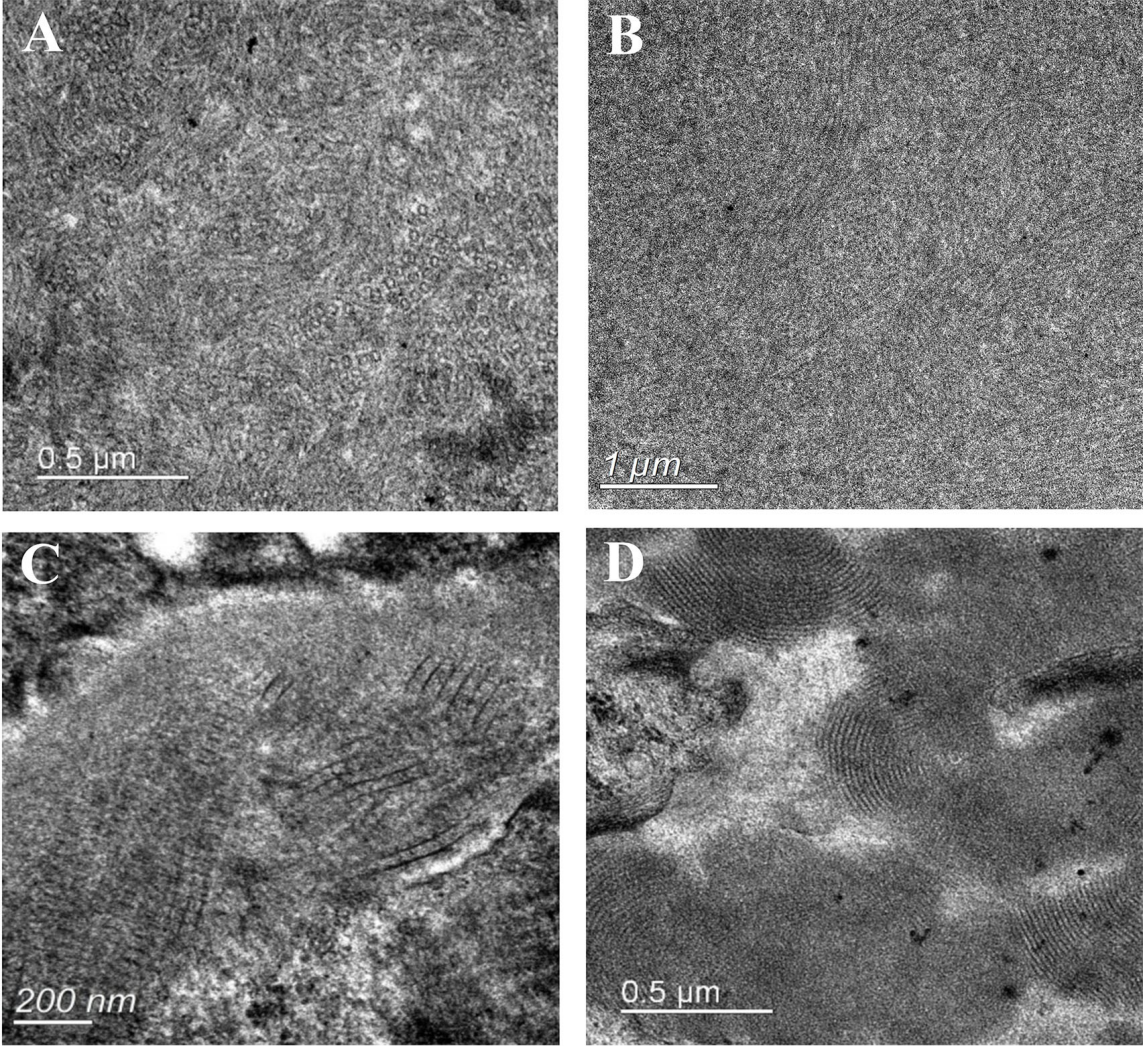
Electron microscopy analysis showing deposits with organized substructure. **(A)** Substructure of curved microtubules in 30-40nm diameter; **(B)** Substructure of fibers with a diameter of 14-30nm; **(C)** Fibers are arranged in a herringbone or grid pattern, with fiber diameters of 6-12nm; **(D)** Substructure of fingerprint.

Sixty-three patients underwent electron microscopy examination, but glomeruli were not obtained in the electron microscopy kidney tissue sections of 6 patients. Electron dense deposits were found in subendothelial, subepithelial, mesangial areas and intracapillary lumens. Organized structures were observed in 17 cases, among which 10 had curved microtubular structures with a diameter of about 10–49 nm (shown in **Figure 3A**), 4 had fibrils about 6–30 nm in diameter (shown in **Figures 3B, C**), 2 had fingerprint structures (shown in **Figure 3D**), and 1 had hollow lattice substructure curved at both ends with some concentric circle-like cross sections.

#### Results of immunohistochemical and multiplex immunofluorescence staining

**Figure 4:**
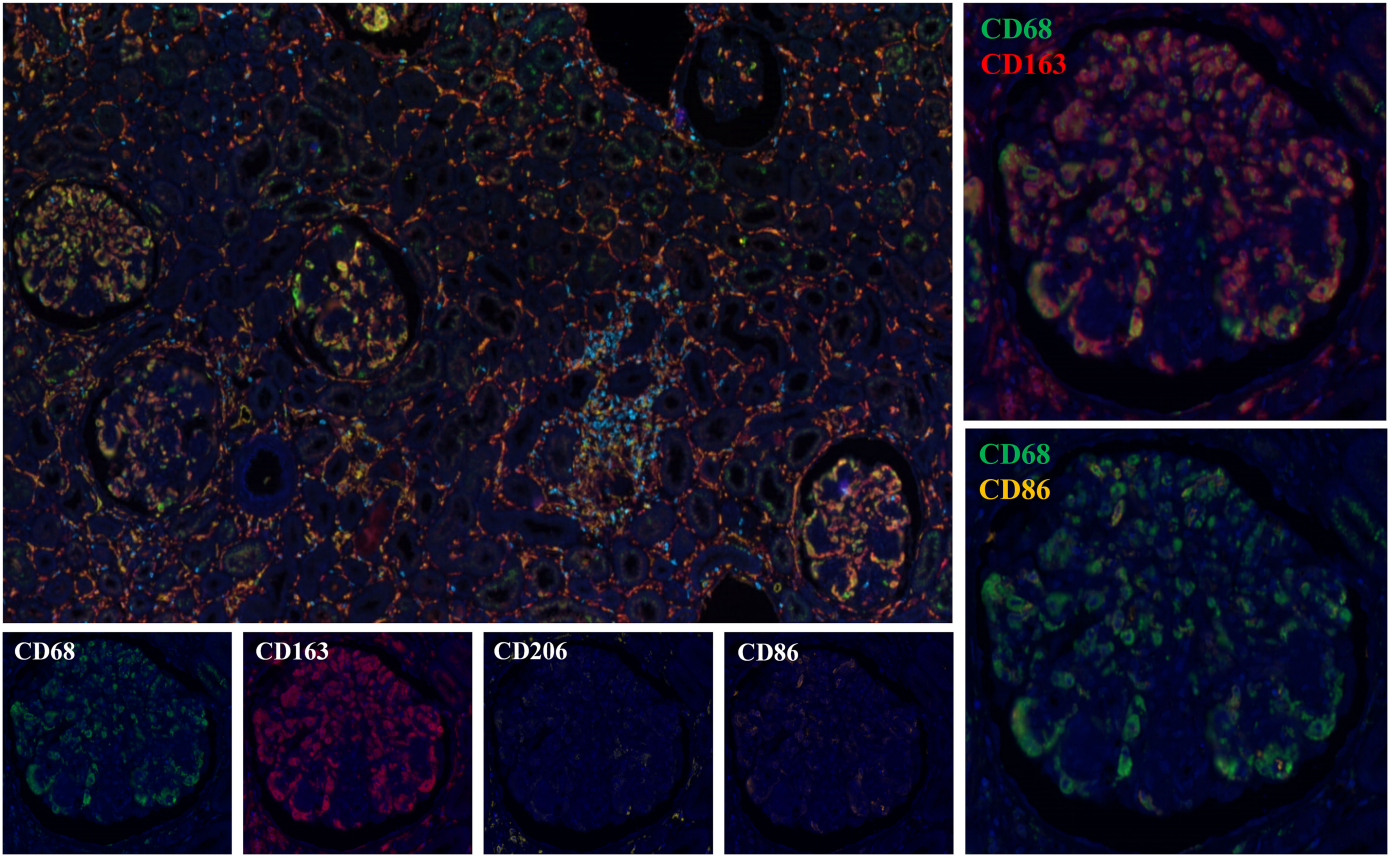
Multiplex immunohistochemical staining analysis results (**Figures 2**, **4** are from the same patient).

We performed immunohistochemical staining for CD68, CD3, CD4, and CD8 on 29 cases. The majority of Cryo-GN patients exhibited a significant infiltration of CD68+ cells within the glomeruli, with a median count of 38 (15–59)/glomeruli (**Supplementary Figures 2A, B**). There were no significant differences in the number of infiltrating macrophages within the glomeruli among the four groups (p=0.107, Highest; p=0.281, Average). Besides, only seven patients demonstrated a sparse infiltration of CD3+ cells within the glomeruli. We selected one case from each of the four groups for multiple immunofluorescence staining, and the results showed that the infiltrating cells within the glomeruli in Cryo-GN were predominantly CD68+CD163+ cells, with a small number of CD68+CD206+ cells and CD3+ cells infiltrating, and CD68+CD86+ cells were rarely observed (**Figure 4**).

### Treatment and renal outcome

The treatment and renal outcome are shown in **Table 3**. Fifty-one patients (73.9%) received immunosuppressive therapy (steroids with or without cytotoxic drugs), 9 patients (13.0%) received clonal-targeted therapy (bortezomib or CD20 monoclonal antibody therapy), and 9 patients (21.7%) received only supportive therapy (including blood pressure control, sodium restriction, renin-angiotensin system (RAS) inhibitors, antiviral or antibacterial therapy when necessary). Forty-seven patients (68.1%) received RAS inhibitors, while 10 patients (14.5%) with HBV and 5 patients (7.2%) with HCV received antiviral therapy.

**Table 3:** Treatment and outcome in Cryo-GN patients without autoimmune diseases.

Treatment and outcome parameters	All patients (n=69)	MIg (n=15)	HBV/HCV (n=18)	MIg+HBV/HCV (n=10)	MIg/HBV/HCV negative (n=26)	P value
Treatment regimen						
Immunosuppressive therapy	51 (73.9)	10 (66.7)^cef^	11 (61.1)	6 (60.0)	24 (92.3)	0.035
Clone-targeted therapy	9 (13.0)	4 (26.7)	2 (11.1)	1 (10.0)	2 (7.7)	0.414
Supportive Therapy	9 (13.0)	1 (6.7)^ef^	5 (27.8)	3 (30.0)	0 (0.0)	0.006
RAS inhibitor	47 (68.1)	9 (60.0)	14 (77.8)	7 (70.0)	17 (65.4)	0.713
Antiviral therapy	15 (21.7)	0 (0.0)	8 (44.4)	7 (70.0)	0 (0.0)	—
Decline rate of eGFR, after first-line therapy, mL/min/1.7 m2/month#	-4.9 (-34.2-23.2)	-1.3 (-30.4-26.4)	-4.7 (-30.8-45.3)	-43.2 (-63.1-54.1)	-5.0 (-30.2-14.3)	0.690
Decline rate of proteinuria after first-line therapy, g/24 h	-51.6 (-76.7-9.3)	-45.2 (-74.6–21.9)	-38.1 (-64.9-84.2)	-15.2 (-86.1-31.5)	-56.7 (-86.0-25.2)	0.629
Kidney outcome#						
CR	3/67 (4.5)	0 (0.0)	0 (0.0)	1/9 (11.1)	2/25 (8.0)	0.418
PR	19/67 (28.4)	5/15 (33.3)	7/18 (38.9)	1/9 (11.1)	6/25 (24.0)	0.456
NR	29/67 (43.3)	3/15 (20.0)	10/18 (55.6)	3/9 (33.3)	13/25 (52.0)	0.143
ESRD	16/67 (23.9)	7/15 (46.7)^a^ ^c^ ^d^ ^f^	1/18 (5.6)	4/9 (44.4)	4/25 (16.0)	0.012

a MIg vs HBV/HCV, *P*<0.05.

c MIg vs MIg/HBV/HCV negative, *P*<0.05.

d HBV/HCV vs MIg+HBV/HCV, *P*<0.05.

e HBV/HCV vs MIg+HBV/HCV negative, *P*<0.05

f MIg+HBV/HCV vs MIg/HBV/HCV negative, *P*<0.05. #Two patients were lost to follow-up, four patients reached the endpoint at biopsy, and two patients lacked specific examination data, with a total of 61 patients included in the statistical analysis.

**Figure 5:**
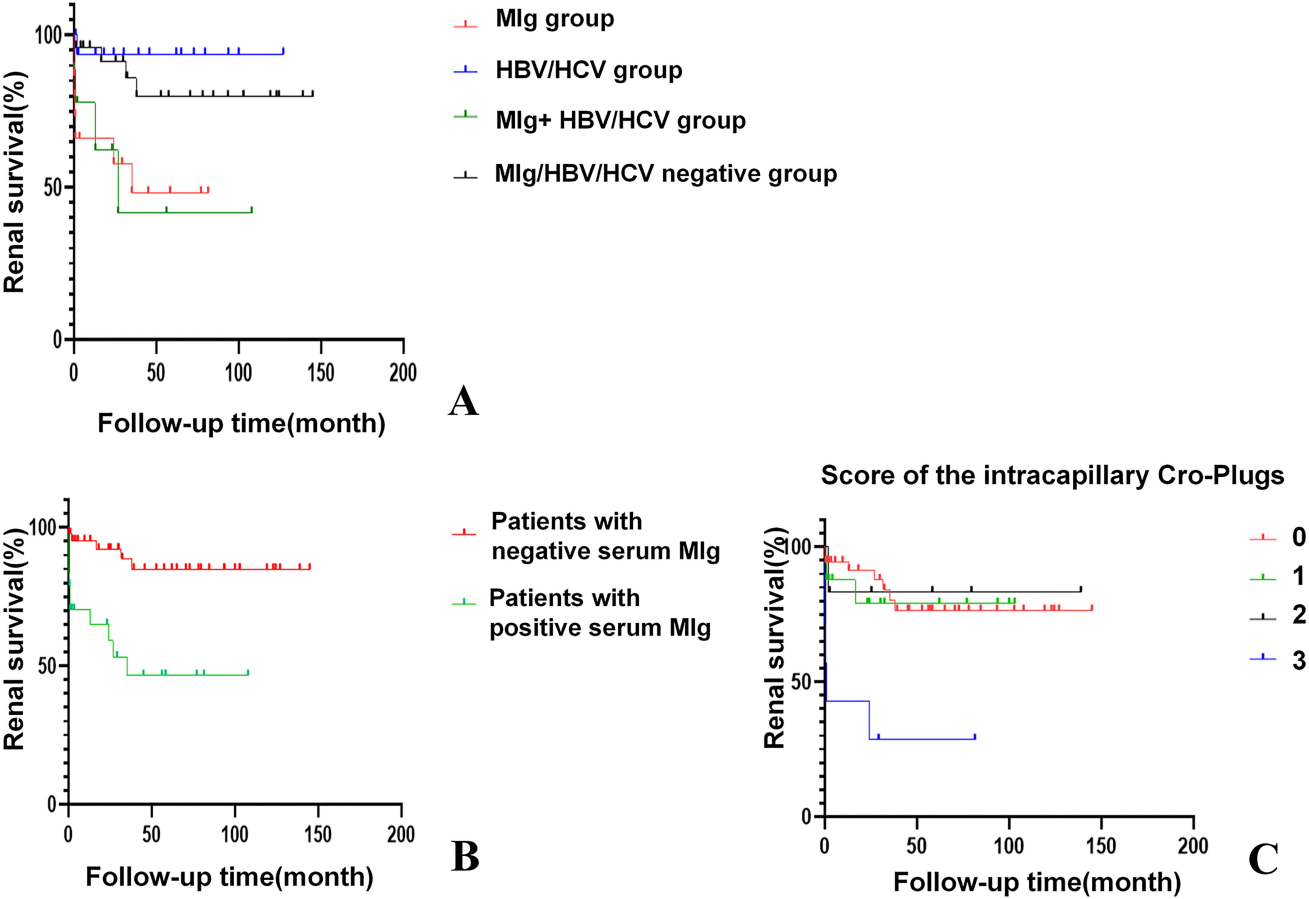
Kaplan-Meier curves of renal survival for Cryo-GN patients. **(A)** Renal survival in patients of different groups. (p=0.007); **(B)** Patients with positive serum MIg had worse renal outcomes. (p<0.001). **(C)** Patients with an intracapillary cryo-Plugs score of 3 demonstrated poorer renal outcomes. (p<0.001) [The severity of intracapillary thrombi in glomeruli was scored according to the proportion of glomeruli affected: 0 (0), <25% (1); 25-75% (2); >75% (3)].

Two patients were lost to follow-up after renal biopsy. The remaining 67 patients had a median follow-up of 31.7 months (2.6-77.1 months). After the first-line treatment, three out of 67 patients (4.5%) achieved complete remission and 19 patients (28.4%) achieved partial remission. At the end of follow-up, 16 patients (23.9%) reached end-stage renal disease (ESRD), of whom 7 patients (46.7%) were in the MIg group. The serum MIg-positive group and the MIg+HBV/HCV negative group had a significantly worse renal prognosis than the HBV/HCV group (p=0.007)(p=0.010) and the MIg+HBV/HCV positive group (p=0.022)(p=0.023) (**Figure 5A**).

Univariate analysis identified six factors associated with ESRD: positive serum MIg, age, eGFR, intracapillary cryo-plugs score, renal insufficiency, and acute tubular injury score. Variable selection in this study was based on univariate Cox regression, clinical significance prior assumptions, and LASSO regression. By multivariate Cox analysis, positive serum MIg and eGFR were independent factors associated with ESRD at last follow-up (**Supplementary Table 2**). The serum MIg-positive patients had worse renal prognosis than the MIg-negative patients (P<0.001) (**Figure 5B**). Patients with the severity of intracapillary cryo-Plugs score of 3 had worse renal outcomes than those with a score of 0, 1, and 2. (P<0.001) (P=0.002) (P=0.020) (**Figure 5C**).

## Discussion

The clinical characteristics of Cryo-GN are various, mainly characterized by purpura, arthralgia and peripheral neuropathy, which are more common in mixed cryoglobulinemia (2, 4, 16, 17). Renal involvement (renal dysfunction or abnormal urinalysis) can be the only clinical manifestation of cryoglobulinemia (9, 10). Based on kidney biopsy data from our hospital, non-autoimmune diseases related Cryo-GN accounts for a low rate of native kidney biopsy (0.08%). The exceptionally low biopsy prevalence of 0.08% in our cohort starkly contrasts with the 1.2-6% reported in HCV-endemic regions like Italy and France (2, 7, 16). This discrepancy may reflect the potential underdiagnosis of atypical presentations, as most of our cases lacked classical cryoglobulinemia symptoms. These findings advocate for earlier nephrological evaluation in patients with unexplained proteinuria or hematuria.

The causes of Cryo-GN are varied, including infection, autoimmune disease and lymphoproliferative disorders. According to the previous studies, HCV infection is the main cause of mixed cryoglobulinemic vasculitis (MCV) and Cryo-GN (18–20). HBV-related MCV in Western countries is rare, and HBV-Cryo-GN has predominantly been reported in small-sample retrospective studies or case series (21–24). In China, 7.18% of the population under 60 years old carry HBsAg (25), while the incidence of HCV infection in mainland China was only 0.43% (26). Some small-sample cohort studies on HBV-Cryo-GN have been reported in China previously (11, 21, 27). In our study, HBV infection was more common than HCV infection in Cryo-GN patients, which was considered to be related to the high prevalence of HBV infection in China.

In the present study, serum MIg was found in 36.2% of Cryo-GN patients without autoimmune diseases. It is worth noting that 40% of the patients with serum MIg had HBV/HCV infection simultaneously. Moreover, the RF positivity rate was significantly higher in the HBV/HCV infection-related groups (HBV/HCV group and MIg+HBV/HCV group). This observation may reflect specific immunopathological mechanisms involving cryoglobulins of different types. Type I cryoglobulins consist of single monoclonal immunoglobulins (typically IgM or IgG) produced by B-cell proliferative disorders including MGUS, multiple myeloma, lymphoma, and Waldenström’s macroglobulinemia. Type II cryoglobulins contain monoclonal IgM with RF activity bound to polyclonal IgG, while type III comprise entirely polyclonal immunoglobulins. In HBV/HCV-associated cases, viral infection can drive monoclonal expansion of innate B cells that produce IgM with RF activity (23, 28), while RF, as a core component of cryoglobulin complexes, may further exacerbate cryoglobulin deposition (28). In addition, Ishitoku et al. found that MGUS-induced cryoglobulinemic vasculitis may occur even after HCV elimination (29). Although HBV/HCV infection may contribute to monoclonal B-cell proliferation, it’s important to recognize that serum MIg in our cohort could also derive from other lymphoproliferative disorders (30, 31). This distinction is crucial as it impacts therapeutic strategy. Therefore, comprehensive cryoglobulin typing and examination for HBsAg/HCsAg in cryoglobulins remains critical for treatment planning.

The severity of intracapillary cryo-plugs was a critical prognostic determinant in our cohort. In glomeruli, elevated protein concentration and fluctuations in anion concentration can promote cryoglobulin aggregation, leading to their deposition on glomerular membranes and subsequent formation of thrombi within glomerular capillaries (32). Besides, the capillary luminal deposits may be related to the molecular characteristics of IgM, which has a larger pentameric structure and a higher intrinsic viscosity, making it more prone to precipitation than other immunoglobulin subtypes (33). Due to the increased protein concentration caused by ultrafiltration, IgM is more likely to deposit in the glomerular capillary lumens (34, 35). By Kaplan-Meier curve, we found that patients with a large number of pseudothrombi in the glomerular capillary lumens tend to have a shorter renal survival time, indicating that the deposition of cryo-Plugs in the lumen may have a special pathogenic mechanism that further aggravates the renal burden. In clinical practice, we believe that the pathology of massive cryo-plugs in the glomerular capillary lumens should be actively paid attention to.

Interestingly, most patients with positive serum MIg showed polyclonal immunoglobulin deposition in renal tissue, while some patients with negative serum MIg had monoclonal immunoglobulin deposition detected in their renal tissue. Moreover, we validated this result by performing monoclonal antibody-specific staining on paraffin sections from the same kidney tissue. This inconsistency essentially reflects the complex immunopathology of Cryo-GN. Several mechanisms may explain this phenomenon: Firstly, serum MIg could act as type II cryoglobulin components inducing deposition of polyclonal Ig in renal tissue, masking the originally monoclonal components (2, 3, 30). Secondly, HBV/HCV infection can trigger B cell oligoclonal expansion, generating antibodies against similar variable regions in the kidneys, appearing monoclonal-like without detectable serum MIg (27, 28). Finally, the methods for detecting monoclonal immunoglobulins also have limitations. In clinical practice, a comprehensive assessment integrating serological tests (cryoglobulin typing, HCV infection), renal pathological presentations, and clinical manifestations is required, rather than relying on a single indicator.

In this study, the etiology of Cryo-GN patients negative for MIg/HBV/HCV remains incompletely defined, possibly associated with occult lymphoproliferative disorders or clonal plasma cell diseases, and these patients may develop lymphoproliferative diseases during follow-up with detectable monoclonal immunoglobulins. However, other infections (such as EBV, cytomegalovirus, or bacterial infections) or unrecognized inflammatory states may also contribute to the development of cryoglobulinemia in these patients (2, 3). While serum MIg detection typically coincides with the diagnosis of underlying hematological malignancies (e.g., lymphoma, myeloma), certain hematologic tumors may not produce monoclonal immunoglobulins, leading to false-negative serological results (36). Moreover, some studies have shown that after the diagnosis of cryoglobulinemia, there was an increased risk of developing hematologic malignancy, especially non-Hodgkin lymphoma (NHL) (37, 38). Although patients with Sjögren’s syndrome-associated cryoglobulinemic glomerulonephritis were not included in this study, clinicians should remain vigilant about the potential risk of lymphoma in patients with Sjögren’s syndrome-associated cryoglobulinemia (39). The identification of monoclonal cryoglobulins (type I or type II with monoclonal IgM-RF) mandates a thorough evaluation for underlying hematological malignancies, including serum/urine immunofixation electrophoresis, serum free light chain assay, and bone marrow biopsy. This is critical because type I cryoglobulinemia is invariably associated with clonal lymphoproliferation (e.g., WM, lymphoma), and even type II cryoglobulinemia may reflect occult B-cell dysregulation in virus-negative cases.

Under electron microscopy, ultrastructure of curved microtubules and fibrils was observed in some Cryo-GN patients, and it is significant for the differential diagnosis of Cryo-GN with fibrillary glomerulonephritis and immunotactoid glomerulopathy (40, 41). It is found that electron-dense deposits with ultrastructure were more common in patients with serum MIg or monotypic IgG deposition in kidney, which indicated that monoclonal Ig-related cryoglobulin may be more likely to form ultrastructure. Previous studies also showed monoclonal cryoglobulins were easy to form ultrastructure, often presenting with microtubule substructures, crystal/crystalloid substructures with parallel arrangement of the filaments, etc. (41, 42).

Macrophage infiltration in the glomeruli is a distinctive feature of cryoglobulinemic MPGN, which was also confirmed in this study. Guo et al. have confirmed that macrophages are essential contributors to kidney injury in murine cryoglobulinemic MPGN (43), where they observed high expression of M1 macrophage-related markers. Meanwhile, M2 macrophages with marker of CD206 were also found in the majority of glomeruli. Differently, we found less CD68+CD86+ cells in the glomeruli, a large number of CD68+CD163+ cells in the glomeruli, and a large number of CD68+CD206+ cells in the interstitium of Cryo-GN patients through multiple immunohistochemical staining. Previous studies have found that CD163+ and CD206+ cells can promote matrix proliferation and interstitial fibrosis in chronic kidney disease, and M2 macrophages are associated with the formation of crescents in IgA nephropathy and the chronicity of the disease (44, 45). We speculate that the disease has entered a chronic phase at the time of renal biopsy in our patient corhort.

Due to the complexity of Cryo-GN, the treatment of Cryo-GN is various according to associated disease. For the treatment of Cryo-GN associated with lymphoproliferative disorders, chemotherapy was adopted for hematopoietic malignancies that producing cryoglobulin or non-malignant proliferative MGRS: the plasma cell derived (monoclonal IgG) were treated with anti-plasma cell drugs such as bortezomib, and/or thalidomide, lenalidomide; lymphoplasmacytoid cell lymphoma can produce monoclonal IgM and often take the rituximab(RTX)-containing treatments (46). Antiviral therapy should be employed in patients with HBV or HCV infection-related Cryo-GN. In addition, immunosuppressants (corticosteroids, CTX, etc.) are important for the treatment of cryoglobulinaemic vasculitis. Studies have shown that RTX combined with antiviral drugs or prednisone is more effective than antiviral or corticosteroids alone, and patients can achieve higher and faster renal complete remission (47–49).

It’s reported that around 10% of the Cryo-GN patients would progress to ESRD (7–11). Previous studies on Cryo-GN showed a large variation in the rate of progression to ESRD due to different follow-up time and etiologies, and identified different independent risk factors for ESRD (**Table 4**). The differences in renal injury among various types of cryoglobulinemia are determined by the composition of immune complexes and the degree of complement activation: Type I shows direct monoclonal immunoglobulin deposition with minimal inflammation; Type II involves monoclonal IgM-polyclonal IgG complexes causing strong complement activation and membranoproliferative glomerulonephritis; Type III features polyclonal immune complexes producing milder mesangioproliferative lesions (2, 50, 51). By grouping the patients according to the presence or absence of serum MIg and HBV/HCV infection, we can better reveal the impact of HBV/HCV infection and serum MIg on the development and prognosis of Cryo-GN. Multivariate Cox analysis revealed that serum MIg and eGFR are independent risk factors for the renal prognosis of Cryo-GN. Coliche et al. also found that elevated cryglobulin concentration and IgG κ monoclonal components were independent predictors of renal involvement (6). This suggests that we should pay more attention to the relationship between serum MIg and Cryo-GN in clinical practice. However, we think further studies are also needed to reveal the role of serum MIg in the occurrence and development of Cryo-GN.

**Table 4:** Clinicopathologic features and outcome in this study and previous reports.

Clinicopathologic features and outcome parameters	Tarantino (7) (n=105)	Roccatello (8) (n=146)	Matignon (9) (n = 20)	Zaidan (10) (n = 80)	Xin Zhang (11) (n=74)	This study (n = 69)
HCV infection, n (%)	29 (85)	129 (88)	0	0	4 (5.4)	7 (10.1)
Hematological malignancy, n (%)	0	ND	1 (5)	23 (28.7)	6 (8.1)	7 (10.1)
Autoimmune disease, n (%)	0	ND	9 (45)	18 (22.5)	0	0
Age at diagnose (years)	52.7 ± 10.65	52.2 ± 13	60 ± 12	62.6 ± 14.1	52.9 ± 15.0	50.4 ± 14.7
Woman (%)	59	56.8	60	62.5	28.4	58.0
Hypertension (%)	82	55	80	85.3	73.0	59.4
Decrease of C4 level (%)	ND	>76	95	75.4	31.1	44.9
Decrease of C3 level (%)	ND	>41	10	ND	ND	68.1
Renal insufficiency (%)	47#	58	85	82.3	ND	60.9
Microscopic hematuria (%)	55	88	100	97.4	90.5	94.2
Nephrotic proteinuria (%)	20	21	75	49.4	52.7	44.9
eGFR<60 (%)	ND	56	85	82.3	ND	60.9
MPGN (%)	80	80	100	92.5	70.3	90.0
Time of follow-up (months)	72 (1-283)	97.2 (average)	48 (3-264)	49.9 ± 45.5	24 (1-23)	31.7 (2.6-77.1)
Independent risk factors for ESRD	Age, purpura, splenomegaly, serum levels of C3, Scr, cryocrit.	Age, serum levels of Scr and proteinuria	ND	Dialysis at diagnosis, extracapillary proliferation	eGFR <45 mL/min/1.7 m2	Positive serum MIg and eGFR
Progression to ESRD (%)	14.3*	11.1¥	10	9	24.3	23.9

#, increase of serum creatinine levels; *, chronic renal functional failure, serum creatinine levels>4mg/dL; for at least six consecutive months; ¥, including 5 patients on dialysis therapy; ND, no data.

The study has limitations. Firstly, it is a retrospective study, so the analysis is hampered by incomplete data, short follow-up time, and unstandardized therapy. Secondly, some patients lacked IgG subtype staining due to the unavailability of kidney biopsy samples for immunofluorescence (IF) examination. Finally, the number of cases for multiplex immunohistochemical staining is relatively limited.

## Conclusion

In conclusion, whether or not there is a HBV/HCV infection, patients with serum MIg in Cryo-GN have worse renal function and outcomes, and patients with a large number of pseudothrombi in the glomerular capillary lumens tend to have a shorter renal survival time; serum immunofixation electrophoresis and immunoglobulin typing in kidney samples are important for finding the cause and guiding the treatment.

## Data Availability

The original contributions presented in the study are included in the article/**Supplementary Material**. Further inquiries can be directed to the corresponding author/s.
