# Supplementary material for: Renal prognostic value of serum monoclonal immunoglobulin in cryoglobulinemic glomerulonephritis
Source: Front Immunol. 2025 Jul 29;16:1578295. doi: 10.3389/fimmu.2025.1578295 (PMC12339349; doi:10.3389/fimmu.2025.1578295)
Supplement: Supplementary file 1 [file DataSheet1.pdf]

Supplementary Material

1 Supplementary Figures and Tables

Supplementary Table 1. Immunofluorescence of patients with serum MIg and/or single Ig or IgG subtype deposits in renal tissue.

Groups(n)	No.	SIFE results	Glomerular Ig deposits	IgG subtype	Light-chain deposits	Organized deposits on EM	Results of bone marrow biopsy
**MIg (n=15)**	1	IgGκ	IgG IgM C3	IgG1, IgG3	κλ	No	N
	2	IgMκ	IgM C3	—	κ	Curved microtubules	Small B cell lymphoproliferative disorder
	3	IgGκ	IgG IgM C3	IgG3	κ	Special structure*	N
	4	IgGλ	IgG IgA IgM C3 C1q	IgG1	κλ	Packedfibrils	N
	5	IgMκ	IgG IgM C3 C1q	IgG1, IgG2	κλ	Curved microtubules	N
	6	IgMκ	IgG IgA IgM C3	ND	κλ	No	Small B-cell lymphoma
	7	κ	IgG IgM C3	IgG1, IgG3	κλ	ND	N
	8	IgMκ	IgG IgM C3	IgG1-4	κ	Curved microtubules	N
	9	IgMκ	IgG IgA IgM C3	IgG1-4	κλ	No	Hypoplasia of hematopoietic tissue
	10	IgMκ	IgG IgA IgM	IgG1, IgG3	κλ	ND	N
	11	IgGλ	IgG IgA IgM C3 C1q	IgG1-4	κλ	No	ND
	12	IgMκ	IgG IgM C3 C1q	IgG1-4	κλ	No	N
	13	IgGλ	IgG C3	ND	λ	ND	N
	14	IgMk	IgM C3 C1q	ND	κλ	Disordered array of fibrils	B-lymphoproliferative disorder

	15	IgMκ	IgG IgA IgM C3 C1q	IgG1-4	κλ	No	B-lymphoproliferative disorder
**MIg+HBV/HCV (n=10)**	16(HCV)	IgMκ	IgM C3	—	κ	No	ND
	17(HCV)	IgMκ	IgG IgA IgM C3	ND	ND	No	ND
	18	κ	IgG IgM C3	IgG1-4	κλ	No	N
	19	IgGκ	IgG IgA IgM C3	ND	ND	ND	N
	20	IgMκ	IgG IgM C3	ND	κλ	No	N
	21	IgMκ	IgG IgM C3	IgG1-4	κλ	No	N
	22	λ	IgG IgA IgM C3 C1q	IgG1-4	κλ	No	ND
	23	IgGκ	IgG IgA IgM C3 C1q	IgG1-4	κλ	fingerprint structure	ND
	24	IgMκ	IgG IgA IgM C3 C1q	IgG1-4	κλ	No	N
	25	IgMk	IgG IgM C3 C1q	IgG1	κλ	Curved [illegible]	N
**HBV/HCV** **（n=5）**	26(HCV)	—	IgM C3 C1q	—	κλ	ND	ND
	27(HCV)	—	IgM C3	—	κλ	No	Small B cell lymphoproliferative disorder
	28(HBV)	—	IgM C3	—	κ	Packed fibrils	N
	29(HBV)	—	IgM C3	—	κλ	No	N
	30(HBV)	—	IgG	IgG1	κλ	ND	ND
**MIg/HBV/HCV**	31	—	IgG C3 C1q	IgG3	κλ	No	N
**negative (n=4)**	32	—	IgG IgM C3 C1q	IgG3	κλ	No	N
	33	—	IgG IgM C3	IgG3	κ	No	N
	34	—	IgM C3	—	κλ	ND	Small B-cell lymphoma

SIFE：serum immunofixation electrophoresis. Ig: immunoglobulin. EM：electron microscope. ND：IgG subtype was not performed or no data. *：deposits were identified as hollow lattice structure curved at both ends, some cross sections were concentric circle like. N：no abnormalities.

Supplementary Table 2. Cox analysis of variables predicting renal survival.

Parameters	Univariate analysis		Multivariate analysis	
	P value	HR（95%CI）	*P* value	HR（95%CI）
**Age**	0.021	1.047(1.007-1.088)		
**Renal insufficiency**	0.050	3.509(0.998-12.336)		
**Positive serum MIg**	0.002	5.249(1.815-15.180)	0.025	3.384(1.114-10.277)
**Decrease of C3 level**	0.800	0.877(0.318-2.417)		
**eGFR**	0.002	0.961(0.938-0.985)	0.002	0.967(0.943-0.992)
**Nephrotic proteinuria**	0.320	1.053(0.951-1.167)		
**Urine NAG**	0.219	1.006(0.997-1.015)		
**Globally sclerotic glomeruli (%)**	0.321	6.063(0.173-212.9)		
**Score of Acute tubular injury**	0.036	1.795(1.038-3.105)		
**Score of the intracapillary Cryo-Plugs**	0.004	1.885(1.224-2.905)		

TA/IF: tubular atrophy and interstitial fibrosis. EPV=8 is slightly lower than the recommended value of 10, model goodness-of-fit is good, but results interpretation still requires caution.

1.1 Supplementary Figures

Supplementary Fig 1. Deposition of multiple heavy-and light-chain isotypes was detected in one patient with Cryo-GN on immunofluorescence.

Immunofluorescence study showed granular staining of IgG (++), IgM (++), C1q (+), IgG1(++), IgG2 (++), IgG4 (+), κ light chain (++), and λ light chain (++) in the glomerular mesangium, capillary walls, and capillary lumens (×400).

Supplementary Fig 2A: Highest CD68 cell count in glomeruli.

Supplementary Fig 2B: Average CD68 cell count in glomeruli.
