# Supplementary figures and images for: Renal prognostic value of serum monoclonal immunoglobulin in cryoglobulinemic glomerulonephritis
Source: Front Immunol. 2025 Jul 29;16:1578295. doi: 10.3389/fimmu.2025.1578295 (PMC12339349; doi:10.3389/fimmu.2025.1578295)

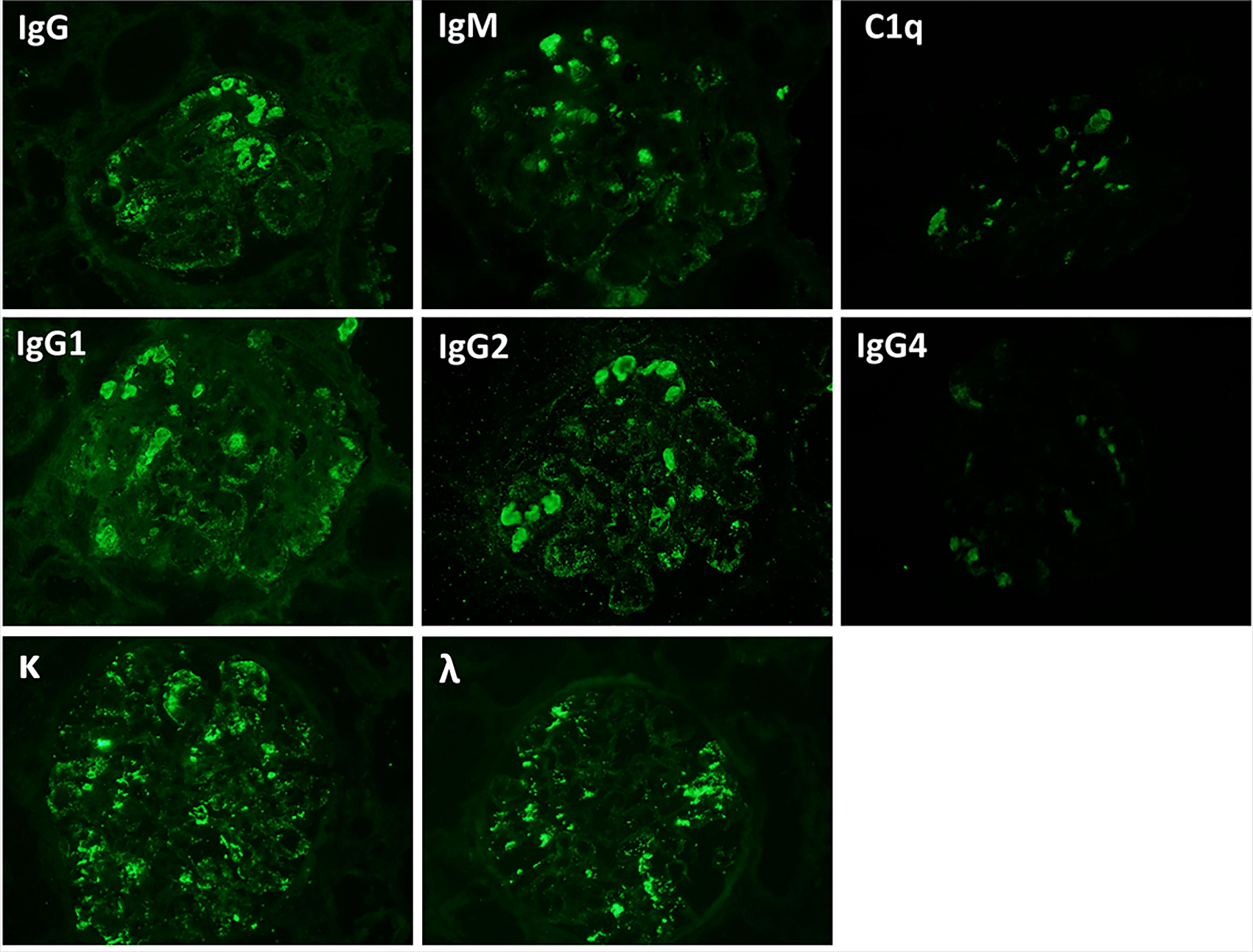

Supplement: Supplementary file 2 [file Image1.tif]

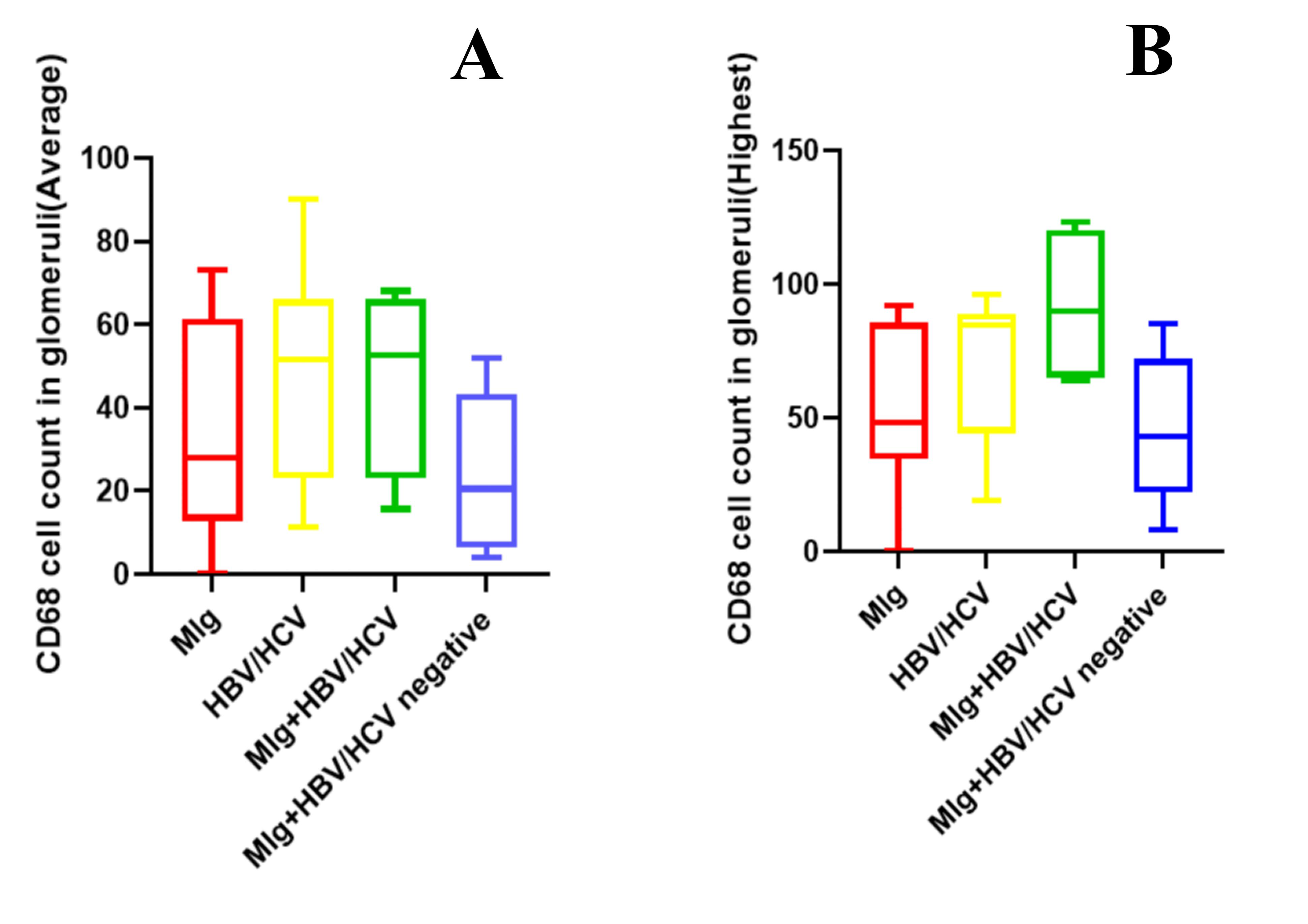

Supplement: Supplementary file 3 [file Image2.tif]
